# Early-Life Environment Influence on Late-Onset Alzheimer’s Disease

**DOI:** 10.3389/fcell.2022.834661

**Published:** 2022-02-17

**Authors:** Thibaut Gauvrit, Hamza Benderradji, Luc Buée, David Blum, Didier Vieau

**Affiliations:** ^1^ Inserm, CHU Lille, U1172 LilNCog—Lille Neuroscience and Cognition, Université de Lille, Lille, France; ^2^ Alzheimer and Tauopathies, LabEx DISTALZ, Lille, France

**Keywords:** Alzheimer disease, perinatal environment, epigenetic mechanisms, early-life stress, tau, amyloid-β

## Abstract

With the expand of the population’s average age, the incidence of neurodegenerative disorders has dramatically increased over the last decades. Alzheimer disease (AD) which is the most prevalent neurodegenerative disease is mostly sporadic and primarily characterized by cognitive deficits and neuropathological lesions such as amyloid -β (Aβ) plaques and neurofibrillary tangles composed of hyper- and/or abnormally phosphorylated Tau protein. AD is considered a complex disease that arises from the interaction between environmental and genetic factors, modulated by epigenetic mechanisms. Besides the well-described cognitive decline, AD patients also exhibit metabolic impairments. Metabolic and cognitive perturbations are indeed frequently observed in the Developmental Origin of Health and Diseases (DOHaD) field of research which proposes that environmental perturbations during the perinatal period determine the susceptibility to pathological conditions later in life. In this review, we explored the potential influence of early environmental exposure to risk factors (maternal stress, malnutrition, xenobiotics, chemical factors … ) and the involvement of epigenetic mechanisms on the programming of late-onset AD. Animal models indicate that offspring exposed to early-life stress during gestation and/or lactation increase both AD lesions, lead to defects in synaptic plasticity and finally to cognitive impairments. This long-lasting epigenetic programming could be modulated by factors such as nutriceuticals, epigenetic modifiers or psychosocial behaviour, offering thus future therapeutic opportunity to protect from AD development.

## Introduction

Alzheimer disease (AD), for which only symptomatic treatments are currently available, is a chronic neurodegenerative disorder and the most prevalence form of dementia. In 2020, there were over 50 million people worldwide living with dementia and this number is estimated to double every 20 years (World Health Organization, 2021). AD is a major health problem characterized by the progressive and irreversible development of neuronal damages, leading to the decline in cognitive functions, notably memory. AD, whose diagnosis is firmly established post-mortem, is defined by the extracellular accumulation of amyloid -β (Aβ) peptides into extracellular amyloid plaques (Checler, 1995) and the presence of neuronal neurofibrillary tangles (NFT) made up of intraneuronal fibrillar aggregates of hyper- and/or abnormally phosphorylated Tau proteins (« Tau pathology »), a common feature of other neurodegenerative disorders called tauopathies (Sergeant et al., 2008). In AD, spreading of the neurofibrillary lesions in the cortex (first enthorinal cortex, then hippocampus and lastly neocortex) fits to the progression of the symptoms, supporting that Tau pathology is indeed instrumental in cognitive alterations (for review Colin et al., 2020). In agreement, Tau pathology development impairs various forms of synaptic plasticity and cognitive behaviors in mouse models (Polydoro et al., 2009; Hoover et al., 2010; Van der Jeugd et al., 2011; Burnouf et al., 2013; Lo et al., 2013; Van der Jeugd et al., 2013). In most cases, AD appears as a multifactorial disease resulting from the complex interaction between genetic predisposition and epigenetic environmental factors (Reitz et al., 2011; Armstrong, 2019). Epidemiological studies identified protective factors such as exercise, intellectual activities and consumption of fish or caffeine (La Rue, 2010; Flaten et al., 2014; Najar et al., 2019). By contrast, life-stresses, preceding clinical manifestation of AD, drive the progression of the disease and can exacerbate symptoms (Reitz and Mayeux, 2009; Reitz et al., 2011). In addition, apart from aging itself, cardiovascular dysfunctions such as obesity, hypertension and diabetes have been suggested as important risk factors for AD (Craft et al., 1998; Tortelli et al., 2017; Flores-Dorantes et al., 2020; Livingston et al., 2020; Tini et al., 2020). It is also notable that AD patients have been reported to exhibit altered glucose metabolism (Bucht et al., 1983; Craft et al., 1992, Craft et al., 1998; Matsuzaki et al., 2010) and even present an increased prevalence to develop type 2 diabetes (Janson et al., 2004). Presence of cognitive and metabolic alterations in AD patients fits with the “Type 3 diabetes” concept, stating that AD brain is insulin-resistant (Talbot et al., 2012; de la Monte, 2014; de la Monte et al., 2018; Nguyen et al., 2020). Although the pathophysiological mechanisms linking AD, insulin resistance and impaired glucose homeostasis remain to be clarified, it has been reported that Aβ oligomers impair insulin signalling by promoting insulin neuronal receptor internalization (Zhao et al., 2008) and inhibit insulin receptor substrate (IRS) 1 (Bomfim et al., 2012). In addition, using Tau knock-out mice, we and others showed that Tau loss-of-function, which is likely a consequence of Tau aggregation, might contribute to brain insulin sensitivity and metabolic impairments (Marciniak et al., 2017; Wijesekara et al., 2018; Wijesekara et al., 2021); an effect reversed by human Tau re-expression (Wijesekara et al., 2021). In line, the H1 Tau haplotype was associated to the glucose homeostasis in humans (Marciniak et al., 2017).

Interestingly, the pioneering epidemiological studies of David Barker in the early 90s established a relationship between intra-uterine-growth retardation (IUGR) and cardiovascular diseases in adulthood paving the way to a new field of research now known as DOHaD (for Developmental Origin of Health and Diseases) stating that environment during early life may shape the rest of our life (Barker, 2007). In accordance, numerous epidemiological studies in humans and experimental works in animals have clearly reinforced the idea that adverse perinatal environment, such as maternal stress, exposure to toxics and early-life malnutrition, has long-term consequences and may program, in the offspring, chronic adult diseases such as glucose dyshomeostasis and energy metabolism impairments but also cognitive disorders (Moody et al., 2017; Gawlińska et al., 2021b), all being encountered in AD patients. In light of these observations, altered perinatal neurodevelopment might sensitize to the occurrence of late-onset AD (Moceri et al., 2000; Landrigan et al., 2005; Miller and O’Callaghan, 2008; Modgil et al., 2014; Athanasopoulos et al., 2016) presumably *via* epigenetic mechanisms (Gapp et al., 2014; Lemche, 2018). In this review, we will summarize the most recent data suggesting that AD, that is usually associated with metabolic impairments developing at the adult stage and aging, may also date back to very early life. The identification of early mechanisms linking parental environment with modification of brain’s epigenetic landscape in the offspring may offer new therapeutic strategies to prevent the progression of AD and other neurodegenerative diseases.

## Tau and AβPP During Early-Neurodevelopment

Maturation of central nervous system (CNS) and establishment of neuronal connections that vary in a region-specific manner can be divided schematically in two major developmental time-periods. The first period that occurs predominantly in the embryonic developmental stages in rodents and within the first two trimesters in humans, is dedicated to neurogenesis, neuronal migration and polarization whereas the second period covering lactation in rodents and the last trimester of gestation in humans is involved in intense neurite stabilization, synapse formation and establishment of brain circuitry (de Graaf-Peters and Hadders-Algra, 2006; Metzger, 2010; Cisneros-Franco et al., 2020). This indicates that the perinatal period covering both gestation and lactation constitutes a particularly sensitive critical time-window during which environmental perturbations may modify brain circuitry and may thus exert long-lasting effects on brain function, synaptic plasticity, homeostasis regulation and cognitive functions. Although the role of Tau and the metabolism of amyloid-β precusor protein (AβPP), the precursor to Aβ peptides, have been extensively described in the context of AD development, few studies have reported their role during early-neurodevelopment. For instance, AβPP acts as a cell adhesion molecule during different steps of neurodevelopment (Sosa et al., 2017) and may thus participate to the migration and maturation of newborn neurons as well as the formation of synapses. *Via* its large ectodomain, AβPP has been shown to bind extracellular matrix (ECM) molecules which play fundamental roles in the formation and maintenance of brain architecture during neurodevelopment and in adulthood (Dityatev et al., 2010; Mouw et al., 2014). Moreover AβPP, *via* dimerization and binding to ECM, participates to the migration and adaptation of newborn neurons to the early environment (Marín et al., 2010; Parsons et al., 2010; Cooper, 2013). Although the developmental role of Tau remains poorly understood, Tau is highly expressed in both rodent and human fetal brains with developmental changes in both splicing and phosphorylation. Resulting from an alternative splicing mechanism, six major isoforms of Tau coexist in the human brain with the presence of either 3 or 4 repeated sequences (named below as Tau-3R or Tau-4R) known as the microtubule-binding regions (Sergeant et al., 2008). Tau-3R short-isoform, being most abundant in fetal brain, has less affinity with microtubules, and increases neuronal plasticity (Wang and Liu, 2008; Hefti et al., 2018; Hefti et al., 2019). In rats, the total level of Tau in the brain remains stable after birth and slightly increased during lactation with the appearance of larger Tau isoforms from post-natal day (PND) 5 to PND15 and the concomitant decrease of fetal Tau (Yu et al., 2009). Despite Tau knock-out mice showed moderate neurologic alterations (Ikegami et al., 2000; Lei et al., 2012), presumably due to compensatory mechanisms involving other microtubules associated proteins, *in vitro* studies in cultured mouse and hamster neurons suggested that Tau is involved in axogenesis, neurite outgrowth and neuronal circuit formation (Dawson et al., 2001; Sennvik et al., 2007; Biswas and Kalil, 2018). Recently, it has been proposed that Tau phosphorylation at microtubule domain region at early stages could protect the hippocampal circuit from overexcitation in 1-month-old 3xTg mice (a model of AD) by directly interacting with the pyramidal circuitry that spontaneously generates theta oscillations (Mondragón-Rodríguez et al., 2018). These results suggest that Tau phosphorylation at the initial stages of disease progression could represent a compensatory mechanism that mediates neuroprotection against hyperexcitability (Mondragón-Rodríguez et al., 2020). Taken together, adverse environmental outcomes during early-life which might affect AβPP metabolism and/or Tau protein might be prone to influence brain development and circuitry with long-lasting consequences.

## Epigenetic Mechanisms Mediate Long-Term Influence of Environmental Factors

Epigenetic inheritance has been defined as heritable changes in gene expression that are not encoded in the primary DNA sequence. Epigenetic processes can regulate DNA replication and repair, RNA transcription, and chromatin conformation, which influence in turn transcriptional regulation and protein translation. Epigenetic marks are partly inherited (transmission of epigenetic marks from one generation to another) but are also the signature and reflection of exposure to environments throughout life, particularly the early environment during gestation and lactation, which is a very sensitive period to both internal and external factors. Indeed, epigenetic mechanisms represent a means through which environmental factors can leave long-lasting memory of past experiences (Kundakovic and Jaric, 2017; Cirulli et al., 2020; Bellver-Sanchis et al., 2021; Breton et al., 2021). Although epigenetic modifications are often considered stable over time, epigenetic plasticity exists and may mediate adaptation to drastic environmental changes. Epigenetic mechanisms have broad actions but may mainly regulate 3 biological functions: DNA and RNA methylation, chromatin remodelling and expression of non-coding RNAs.

### DNA and RNA Methylation

DNA methylation is one of the most studied and characterized epigenetic marks. It corresponds to the addition, *via* an enzymatic process, of a methyl (CH_3_) group covalently linked to a cytosine residue in position 5 (5-methylcytosine, 5mC). DNA methylation that occurs mainly on palindromic CpG sequences, which can form CpG islands, takes place during early development and does not increase over time (Jang et al., 2017). However, recent studies indicate that DNA methylation can also occur on non-CpG sequences (called CpH, where H is A, C or T), especially in the brain of vertebrates (de Mendoza et al., 2021). Methylation of CpH, unlike CpG, is established *de novo* during the post-natal period, which corresponds to the maturation processes of neurons, and is increased later in life (Guo et al., 2014; Stroud et al., 2017). DNA methylation on CpG usually leads to inhibition of gene expression when it occurs at the promoter region of genes, while it increases transcriptional activity when it occurs in the transcribed regions. Although many studies suggest that CpH methylation is inhibitory, this is still debated (Jang et al., 2017). The addition of the CH_3_ group is catalysed by DNA methyltransferases (DNMTs). There are 3 DNMTs, DNMT3A and DNMT3B being involved in *de novo* methylation, while DNMT1 catalyses the maintenance of methylation during DNA replication. DNA methylation, although quite stable, is a reversible mechanism mediated by the action of Ten-eleven translocation (TET) enzymes. This family of 3 isoforms catalyses the oxidation of 5mC to hydroxymethylcytosine (5hmC), 5-formylcytosine (5fC) and 5-carboxylcytosine (5caC), the last two products can be excised from DNA through base excision repair, thus resulting in the reversion to demethylated cytosine (for review Lacal and Ventura, 2018). Interestingly, the activity of DNMTs and TETs is particularly high during neurodevelopment and the balance between these two families of enzymes is thought to play a crucial role in the dynamic regulation of brain genes involved in development and in cell differentiation (Tao et al., 2018; Cisternas et al., 2019). A recent study reported the methylation patterns of the Presenilin-1 gene (PSEN1), which encodes the catalytic peptide of the gamma-secretase complex (a key enzyme that cleaves the AβPP), in the brain of AD animals and humans. Interestingly, a temporal correlation between dynamic modifications in the PSEN1 CpG and non-CpG methylation patterns and mRNA expression during neurodevelopment and AD neurodegeneration has been reported (Monti et al., 2020). Moreover, several studies have shown that a decrease in TET and DNMT activity is associated with AD and cognitive impairment (Christian et al., 2020; Zhang et al., 2020; Antunes et al., 2021). Although it is likely that 5hmC represents the first step of active DNA demethylation, several data suggest that the 5hmC mark, which is enriched in adult CNS, is an active and independent epigenetic mark promoting the recruitment of factors allowing or not gene expression (Mellén et al., 2012; Shi et al., 2017; Stoyanova et al., 2021). In addition to DNA, methylation can also occur on mRNAs and lncRNAs. In this case, the addition of the methyl group takes place on adenine at position 6 (m6A) and is catalysed by a protein complex including METTL3 and METTL4 that carry the catalytic domain. This post-transcriptional modification of RNA promotes binding to other proteins, allowing pre-miR formation, translation or mRNA degradation. As with DNA methylation, this mechanism is reversible via the action of FTO and ALKBH5 (for review Zhou et al., 2020).

### Chromatin Remodelling

In mammalian cells, histones interact with DNA to form chromatin. There are four types of histones: H2A, H2B, H3 and H4, which are present in duplicate to form an octamer, around which DNA is bound, forming a nucleosome. In the same way as DNA, histones can undergo several post-translational modifications (acetylation, methylation, phosphorylation, ubiquitination … ), altering the structure of chromatin. Histone methylation, via histone methyltransferase (HMT) activity, on the N-terminal tail changes the degree of chromatin compaction, making the DNA accessible or inaccessible to transcription factors, regulating gene expression. Histone methylation can enable or repress gene expression depending on the histones methylated and the amount of methylation. The mechanism is dynamic as histones can be demethylated by histone demethylases (HDMs). Histones can also be acetylated on the lysine residues of the N-terminal tail. The addition of an acetyl group is catalysed by histone acetyltransferase (HAT), while histone deacetylases (HDACs) remove acetyl groups. Acetylation of lysines opens up chromatin, making the chromatin structure transcriptionally active with a greater access of transcriptional activators. In contrast, deacetylation compacts chromatin, preventing the binding of transcription factors, thus inhibiting gene expression (for review Bannister and Kouzarides, 2011).

### Non-coding RNAs

There are two types of non-coding RNAs (ncRNAs) classified according to their size, ncRNAs below 200 nt are defined as “short” (sRNAs) and those above this length as “long” (lncRNAs). NcRNAs are single-stranded and the result of post-transcriptional maturation. Among sRNAs, microRNAs (22–25 nt), the most studied, can assemble to a protein complex and associate by complementarity with RNA segments usually on the 3’ untranslated region, leading to translational repression or mRNA degradation depending on the level of complementarity. The lncRNAs, whose functions are less studied, have genes similar to the genes that code for proteins and have different modes of action by linking to DNA, RNA and proteins. Non-exhaustively, they can inhibit transcription by preventing the recruitment of the pre-initiation complex, regulate other epigenetic modifications by guiding enzymes (HDACs, HATs, HMTs, HATs, DNMTs … ) to their site of action, activate the expression of neighbouring genes, bind to mRNA leading to mRNA degradation, bind to miRNAs limiting the action of the latter, regulate the splicing (for review Hombach and Kretz, 2016).

Although the emergence of Omics technologies has allowed to better characterize epigenetic modifications in the context of AD, these studies, that have been the subject of recent reviews (Monti et al., 2020; Coppedè, 2021; Nikolac Perkovic et al., 2021), are outside the scope of the present manuscript which is focused on the putative influence of the early environment on AD programming.

## Long-Term Consequences of Early Environment on Tau and Aβ

Exposure to environmental agents has been extensively studied as a risk factor for the development of neurodegenerative diseases, such as AD (Killin et al., 2016; Wajman et al., 2018; Olayinka et al., 2019; Rahman et al., 2020). Recent studies suggest that exposure to cafeteria diets, pesticides, nanoparticles, air pollution and heavy metals during the perinatal period may sensitise to the development of AD in the long term, acting on the two lesions of the disease, Tau pathology and amyloid pathology, but also on neuroinflammation, which is considered as culprit component of the disease. Below, we will discuss the recent literature on the effects of perinatal exposure to different factors on Tau and Aβ.

### Early-Life Exposure to Chemical Agents

Exposure to lead (Pb) during early-life has been particularly studied in the context of AD. Indeed, lead pollution is a major public health issue in developing cities. A meta-analysis using data on Pb exposure in Mexico City residents showed high blood Pb levels associated with a 5-point reduction in Intellectual quotient (IQ), and intellectual disability in children aged 0–4 years-old (Caravanos et al., 2014). Although no epidemiological study reports the effects of long-term Pb exposure, experimental studies in rodents (Bihaqi et al., 2014; Gąssowska et al., 2016) and monkeys (Bihaqi and Zawia, 2013) indicate that early exposure to Pb augments the phosphorylation of Tau, and is associated with an increase in the level of the cyclin-dependant kinase 5 (Cdk5), a major Tau kinase that is involved in abnormal phosphorylation of Tau in AD brain (Cruz and Tsai, 2004). In addition to these effects on Tau, studies also showed an increase in AβPP expression and plaque formation in the aging primate brain induced by early Pb exposure (from birth until 400 days of age). That was associated with a decrease in DNMT1 and DNMT3a activity and with a reduction in the acetylation and methylation of certain histones, resulting in a reprogramming of gene expression associated to neurotransmitter, growth factors and signal transduction pathways, suggesting an epigenetic effect (Wu et al., 2008; Bihaqi et al., 2011).

The action of bisphenol A (BPA), an endocrine disruptor used to manufacture polycarbonate plastics, is also well documented. Interestingly BPA, due to its lipophilic nature, is able to penetrate the blood brain barrier and has been found in the placenta, amniotic fluid, blood breastmilk, suggesting that it could act on foetuses/neonates during the perinatal life (Hines et al., 2015). Indeed, epidemiological studies have associated BPA exposure during early life stages with low birth weight and an increased risk of developing metabolic diseases in adulthood (Chevalier and Fénichel, 2015). In mice, maternal BPA exposure disrupts brain function and induces cognitive deficits (Tian et al., 2010) and learning-memory impairment in offspring (Xu et al., 2010). It also augments AβPP levels and Tau phosphorylation via increased activities of GSK3β and CDK5—two major Tau kinases (Sergeant et al., 2008)—but decreases insulin signalling and alters synaptic plasticity in adulthood (Fang et al., 2016; Xue et al., 2020). Prenatal BPA exposure deregulates the offspring hippocampal transcriptome with genes associated with AD, oxidative stress and inflammation (Sukjamnong et al., 2020). In particular, it induces elevation of NF-κB protein and its AD-related target BACE1, a key enzyme involved in AβPP processing, suggesting that prenatal BPA exposure triggers neuroinflammation in the hippocampus and increases the susceptibility of AD through NF-κB. These modifications may explain the cognitive impairments observed in other studies (Negishi et al., 2004; Ryan and Vandenbergh, 2006; Wang et al., 2014), even with a single dose of BPA at PND10 (Viberg et al., 2011). Indeed, *in utero* and neonatal exposure to low dose of BPA may cause deregulation of miRNAs expression, DNA methylation levels and histone modifications (Singh and Li, 2012). A genome-wide analysis of the foetal mouse forebrain epigenome shows that exposure to low doses of BPA modifies the methylation in the promoter-associated CpG islands at several loci, which can alter the brain development (Yaoi et al., 2008). In rat, the exposure to the Di-(2-thyhexyl)-phtalate (DEHP), another endocrine disruptor, during gestation and lactation also increases the phosphorylation of Tau, without modification of AβPP level in hippocampus, and is associated with cognitive impairments in adulthood (Sun, 2014). However, so far no studies have investigated the long-term consequences of early-life BPA on epigenetic changes in the Microtubule Associated Protein Tau (MAPT, the gene encoding Tau) and AβPP genes.

Exposure to xenobiotics, man-made chemicals found in the environment but not endogenously produced, is increasingly common, and their presence in the brain during the perinatal period can alter CNS development. Polybrominated diphenyl ethers 209 (PBDE 209), a combustion inhibitor that spreads in the environment, has been found in breast milk and in consumer products. Studies in animals show that a single exposure to PBDE 209 at PND10, at the time of brain growth spurt in rodent, does not modify the level of Tau but affects CaMKII and synaptophysin levels in the hippocampus (Viberg, 2009), two proteins playing an essential role in neurodevelopment, whose alterations may likely explain impaired spontaneous behaviour seen in adulthood (Johansson et al., 2008). Similarly, exposure to another xenobiotic, the non-dioxin-like polychlorinated biphenyls (NDL-PCB), during lactation in Swiss albino mice led to a more pronounced decrease of the synaptic proteins synaptophysin and PSD95 and impairment of long-term memory when the brain is challenged by the injection of Aβ oligomers (Elnar et al., 2016).

### Early-Life Exposure to High-Fat Diet

Maternal obesity has considerably increased among women of reproductive age in the last decades. Current estimates suggest that by 2025 more than 21% of women in the world will be obese, and in 2014, the estimated percentage of overweight and obesity among pregnant women was 33% in the United States (Poston et al., 2016). Cumulative data suggest that maternal obesity leads to metabolic and cognitive disorders in the offspring (Hasebe et al., 2021), which makes maternal obesity a major public health issue. However, although cognitive and metabolic impairments are two components of AD, the consequences of maternal perinatal high-fat diet on Tau and Aβ have been poorly investigated. A recent study by Di Meco *et al.* reports that a maternal high-fat diet (mHFD with 42% calories from fat) during gestation decreases total Tau and pathological Tau conformation level and increases the synaptic integrity, leading to improved cognitive performance in adult mice (Di Meco and Praticò, 2019). However, it has also been reported that a mHFD (45% fat) during gestation and lactation increases the level of Aβ peptides at the vascular level, and is associated with a modification of the neurovascular environment in the brain of adult mice, which leads to the alteration of the perivascular clearance of Aβ peptides (Hawkes et al., 2015). Although many works have looked at the epigenetic changes induced by mHFD in the programming of peripheral metabolic diseases, very few studies have looked at the consequences in the CNS of the offspring (Gawlińska et al., 2021a). For instance, it has been reported that consumption of a high-fat diet during gestation and lactation in the Wistar rat decreases the global level of histone H4 acetylation in the hippocampus at P50, leading to transcriptional repression (Gonçalves et al., 2017). Interestingly, a diet rich in grape juice has an opposite effect on the level of this epigenetic mark at PND21 and is associated with a beneficial effect on the brain with an antioxidant effect (Gonçalves et al., 2017; Schaffer et al., 2019) which could limit deleterious consequences of mHFD by reducing oxidative stress and acetylcholinesterase activity (Proença et al., 2021). However, to our knowledge, the putative consequences of mHFD on epigenetic changes in MAPT and AβPP genes have never been reported.

### Early-Life Exposure to Tobacco

According to the WHO, more than 1.3 billion people worldwide smoke, about 40% of them being women. In low- and middle-income countries, where the proportion of people who smoke is highest, the prevalence of pregnant women who smoke is 2.6%, but can be as high as 30.8% in Russia (Caleyachetty et al., 2014). The deleterious effects of smoking on offspring are widely accepted and can lead to IUGR and neurodevelopmental changes (Perera et al., 2005; El Marroun et al., 2016). An epidemiological study on 471 individuals in Finland showed that male offspring of mothers who were exposed to cigarette smoke prenatally had more cognitive problems, although these effects were minor or absent in females (Ramsay et al., 2016). In mice, it has been shown that exposure to cigarette smoke during lactation increases the phosphorylation of Tau, and the levels of the 3R form of brain Tau, leading to a deregulation of the 3R/4R ratio of Tau during a critical period of brain development in 4-week-old offspring (La Maestra et al., 2011). Indeed, Tau phosphorylation but also the proportion of each isoform plays an important role in brain development, and particularly during lactation, during which synaptogenesis and axonal growth take place. Although not reported to date, these early changes could lead to neuronal dysfunction and exert long-term consequences.

### Early-Life Exposure to Ionizing Radiation

Humans are routinely exposed to ionizing radiation (IR) from natural sources, and from man-made sources such as nuclear energy. IR is also increasingly used in the medical field in the diagnosis or treatment of diseases, for example in X-ray imaging. For example, in children, for the treatment of brain tumours, exposure at a very young age can also influence the development of CNS. An epidemiological study on 3,094 Swedish males indicated that low-dose IR exposure during early human development can have a negative impact on cognitive abilities during childhood (Hall et al., 2004). An experimental study in mice reported that a single low-dose exposure of IR to PND10 animals deregulates the hippocampal and cortical proteome and transcriptome, impairs synaptic plasticity, reduces neurogenesis and leads to neuroinflammation later in life. Interestingly, miRNAs analysis showed an increased expression of miR-132 and miR-134 repressing the translation of proteins of the Rac1-Cofilin signalling pathway involved in synaptic plasticity and neurogenesis, highlighting a potential epigenetic mechanism (Kempf et al., 2014). Another team using a similar protocol demonstrated an increase in cortical Tau levels at PND11 (i.e. 1 day after exposure) and at 6 months. That was associated with a decline of long-term spatial memory when IR is combined to ketamine exposure, an anaesthesia commonly used to facilitate radiotherapy in children. Although it has not been directly assessed, these long-term consequences on Tau protein levels and cognitive deficits suggested the involvement of epigenetic mechanisms (Buratovic et al., 2014; Buratovic et al., 2018).

### Early-Life Exposure to Asphyxia

Perinatal asphyxia is a defect in blood flow or gas exchange from the mother to the foetus or vice versa, occurring immediately before, during or after birth. It accounts for up to 20 cases per 1,000 births in developing countries, with mortality during the neonatal period of 15–20%, while 25% of survivors develop short- and long-term neurological disorders, associated with neonatal encephalopathy in most cases (Gillam-Krakauer and Gowen Jr, 2021). The most common diagnosis of perinatal asphyxia is addressed by chest X-ray, which, as mentioned in the previous paragraph, can be deleterious, especially in the young individual. Thus, it is important to uncover biomarkers released into the cerebrospinal fluid (CSF) during perinatal asphyxia to avoid X-ray exposure. With this idea in mind, one team evaluated the levels of Tau, pTau and Aβ42 in the CSF of newborn pigs that had undergone a perinatal hypoxia event a few hours after birth. They showed a decrease in the level of Aβ42, with no change in the level of Tau and pTau. They also observed an increase in S100B (Benterud et al., 2015), a glial marker of neuroinflammation which is also increased in CSF of infants with perinatal asphyxia (Massaro et al., 2014). In contrast, an epidemiological study reported that delivery by caesarean, known to limit hypoxia in cases of perinatal asphyxia, was associated with a reduction in cord blood Tau protein levels, indicating that Tau is modulated by blood oxygen concentration (Tunc et al., 2010) although it could not be excluded that Tau variation results from other factors associated with caesarean delivery. However, it is clear that perinatal asphyxia modified Tau, pTau and Aβ42 in the fetus/neonate, it remains to be determined if these early-life changes influence (or not) the development of late-onset AD.

## Long-Term Consequences of Early Environment in Animal Models of AD

Although early-life stressors exposure affects Tau and Aβ peptides levels, very few studies investigated the effects of perinatal perturbations in the pathological context of AD. This section aims at summarizing the literature on the consequences of early environment on the development of AD lesions.

### Early-Life Exposure to Maternal High-Fat Diet

As previously mentioned, mHFD can have long-term deleterious effects on the levels of Tau and Aβ peptides. The consequences of mHFD have also been studied in the 3xTgAD mouse, a model of AD that develops early amyloid pathology and later Tau pathology. A first team reported that the application of mHFD (60% fat) during gestation and lactation impaired short- and long-term spatial memory and increased the number of Tau-positive neurons in the hippocampus without altering Aβ peptides level, suggesting that mHFD exacerbated Tau pathology and may sensitize to the development of AD (Martin et al., 2014). Surprisingly, using the same animal model it has also been reported that mHFD (42% fat) only applied during gestation protects from synaptic dysfunctions and associated cognitive disorders and decreases Tau and amyloid pathology by reducing the amount Aβ40 and Aβ42. This putative protective effect of the maternal diet could result from an increased gene expression of the transcriptional repressor FOXP2, which could be responsible for the decreased gene expression of Tau, CDK5 (a Tau kinase) and BACE-1 (involved in the amyloidogenic pathway) possibly via epigenetic mechanisms (Di Meco et al., 2019). It is important to note that an important limitation of this model is that the mice are homozygous for all 3 mutant alleles, and thus the observed phenotype is the result of the diet given to transgenic dams. Therefore, it is not entirely clear if changes result from the diet effect only or from the convergent impact of the diet and the transgenic phenotype of dams. Using the Tg2576 mouse, another model of AD expressing the human Swedish AβPP mutation and developing only amyloid pathology, it has been reported that a mHFD (45% fat) starting before crossing until weaning increases the level of soluble Aβ and the number of amyloid plaques. It has been suggested that these alterations could modify the extracellular matrix leading to increase Aβ plaques retention within the parenchyma (Nizari et al., 2016). These seemingly contradictory results may be explained by the different amount or types of fat used in the diets and/or by the timing of exposure of mHFD, as well as by the model used regarding amyloid pathology. Although the biological mechanisms that explain the consequences of mHFD on the development of AD are still poorly understood, recent studies suggest an important role for the gut microbiota. Indeed changes in maternal diet during the perinatal period modify both the mother’s and the offspring’s microbiota with maternal transmission both at delivery and through milk intake (Mueller et al., 2015). In mice, perinatal mHFD alters the short-term memory and is associated with a deregulation of the gut microbiota in offspring, which could partly explain the observed phenotype (Sanguinetti et al., 2019). Interestingly, a high-fibre diet in mothers or offspring can restore cognitive deficits in offspring by regulating the composition of bacteria and SCFA metabolites in the long-term (Liu et al., 2021). It has been suggested that early deregulation of the microbiota may participate to cognitive impairment and to the onset of later lesions in the 3xTgAD mouse model (Bello-Medina et al., 2021). Interestingly, addition of bioactive food to the diet restores these effects and modulates the composition of the gut microbiota (Syeda et al., 2018). Together, these studies indicate that the modification of the diets or the addition of probiotics, both of which being able to modulate epigenetic mechanisms, offer the opportunity for identification of new diagnostic biomarkers and treatments (Ji and Shen, 2021).

### Early-Life Exposure to Caffeine

Tea and coffee are two commonly substances consumed in the world, whose main component is caffeine, a psychoactive substance. Numerous epidemiological studies in humans and experimental studies in AD mouse models have shown the beneficial and long-term protective effects of moderate caffeine consumption in adulthood in reducing the development of lesions and related to cognitive impairment, decreasing the risk of developing AD by 65% in humans (Eskelinen et al., 2009; Flaten et al., 2014; Polito et al., 2018). Sustaining epidemiological studies, caffeine treatment of adult mice developing amyloid or Tau demonstrate positive outcomes at the pathological and behavioural levels (Arendash et al., 2006; Arendash et al., 2009; Laurent et al., 2014). In sharp contrast perinatal caffeine, by blocking adenosine A2A receptors, not only exert a detrimental impact on neuronal migration but caffeine receptors themselves strongly impact GABAergic synapse stabilization during synaptogenesis (Gomez-Castro et al., 2021). Further, perinatal caffeine strongly impacts hippocampal excitability and memory at the adult stage. Whether perinatal caffeine modulates the later development of AD pathology or has cognitive consequences remains largely unclear. However, recent data showed that perinatal caffeine consumption starting before gestation until PND15 in a transgenic mouse model of tauopathy, even not modifying the pathological load, leads to earlier long-term memory impairments together with excitatory/inhibitory synaptic drive in adulthood. These results indicate that early-life caffeine may influence brain development and exert long-lasting consequences in a Tau background (Zappettini et al., 2019).

### Early-Life Exposure to Stress

Evidence indicates that chronic stress can participate to the progression of AD and other neurodegenerative disorders. In 3xTgAD mice, chronic social stress in adulthood, by regularly changing the composition of the mice in the cage, decreases BDNF levels and increases amyloid pathology and anxiety, with no change in control animals indicating a greater sensitivity to stress in mutant mice (Rothman et al., 2012). During the perinatal period, although few studies have been reported, Sierksma *et al.* have shown that chronic restraint stress performed during gestation (E1-E7) in APPswe/PS1dE9 mice model of AD induced sexual-dimorphic results in the adult offspring with males exhibiting altered spatial memory, while females have improved spatial memory associated with decreased amyloid load in the dorsal hippocampus (Sierksma et al., 2012). Although these modifications were not associated with changes in DNMT expression nor 5mC and 5hmC levels, other epigenetic mechanisms targeting histones or non-coding RNAs remain to be studied. Using the same AD animal model, it has been shown that repeated maternal separation during lactation, which is also used to induce stress during early-life, increases amyloid deposits in the hippocampus and cortex, and impairs learning and spatial memory in adulthood (Hui et al., 2017). The same experimental protocol increases Tau and amyloid pathology, decreases synaptophysin and PSD95 (two synaptic markers), impairs memory and is associated with hyperactivity of the hypothalamo-pituitary-adrenal (HPA) axis in adult male rats (Martisova et al., 2013). These observations are in agreement with the elevated glucocorticoid levels in adulthood found associated with a higher risk of developing AD, by altering glial function, Tau and amyloid lesions, and synaptic plasticity (Vyas et al., 2016). The first evidence that early-life stress could induce epigenetic programming in the rat brain came from the pioneer studies of Weaver *et al.* who showed that poor maternal care leads to increased methylation in the glucocorticoid receptor (GR) promoter which was accompanied by aberrant behaviours and altered HPA responses in adult offspring (Weaver et al., 2004). Using the 3xTgAD mice, it has been reported that handling offspring during lactation (PND2-PND9), by separating the dam from her pups for 15 min, increases maternal care of the dam towards her pups upon reunion. Increased maternal care leads to a decrease both in short-term memory decline and in amyloid load in the hippocampus, suggesting that prenatal stress could be mitigated or to have antagonistic effect by postnatal maternal care.

Recently, it has been reported in a subclinical AD-like rat model, that adult animals which were prenatally-stressed (PS) exhibited increased dysfunctions of HPA axis activity and working and long-term spatial memory as well as modified cellular organisation in CA1 hippocampal region. Interestingly, these alterations were alleviated by increased postnatal maternal care during lactation. Unfortunately, the authors did not report the putative consequences of cross-fostering in adult PS rats on Aβ plaques and NFT nor on phosphorylation and protein levels of Tau (Rostami et al., 2020).

### Conclusion and Perspectives

**FIGURE 1:**
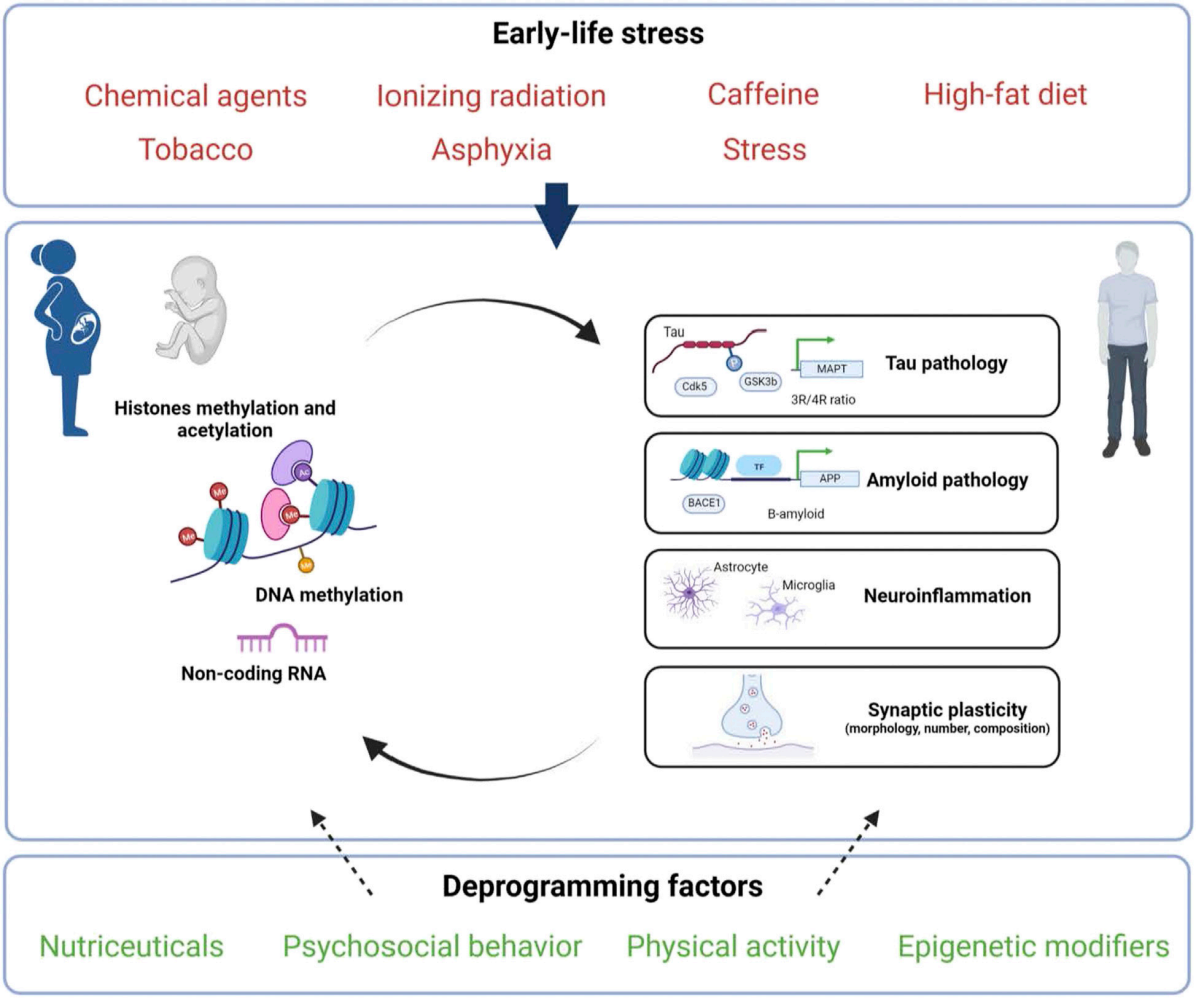
Putative factors and epigenetic mechanisms linking early-life environment to late-onset Alzheimer’s disease programming. Exposure to diverse environmental stressors (indicated in red) during perinatal life (gestation and/or lactation) may influence epigenetic mechanisms (DNA/RNA, histones and/or non-coding RNAs) and thus could have both short- and long-lasting consequences on Tau, A-β peptides, neuroinflammation and/or synaptic plasticity. These early exposures may increase the risk to develop AD but may also be counteracted by deprogramming factors (indicated in green) that could offer novel therapeutic strategies. Created with BioRender.com.

In the present review, we provided strong evidence that early-life stress may constitute another risk factor contributing to the development of late-onset AD possibly *via* long-term influence of epigenetic mechanisms (Figure 1). However, important questions, which are still unanswered in the context of AD, such as the putative contribution of transgenerational epigenetic inheritance, paternal contribution and sexual dimorphism on late-onset AD development, have been overlooked. It should be kept in mind that although epigenetic marks may be transmitted transgenerationally (Burton and Greer, 2021), the existence of multigenerational epigenetic inheritance in humans is still a matter of debate (Cavalli and Heard, 2019). Epigenetic mechanisms are indeed plastic, modifiable and reversible processes providing thus opportunities to try to reverse deleterious effects of environment *via* early-life interventional strategies (Lesuis et al., 2018). Recently, the consequences of mHFD (60% fat) have been examined in F1 (intergenerational inheritance) and F2 (transgenerational inheritance) generations of SAMP8 mice, a model of accelerated ageing leading to increased cognitive decline associated with the development of neuropathological AD hallmarks (Liu et al., 2020). The HFD applied to female mice of the F0 generation was supplemented or not with resveratrol, a natural phytoalexin known to have beneficial effects in pathological conditions, notably via its anti-inflammatory effects, and by reducing amyloid pathology and playing a role in epigenetic mechanisms (Rahman et al., 2020). Interestingly, the impairment of both short- and long-term memory and metabolic parameters (increased plasma levels of triglycerides and leptin) induced by the HFD was attenuated by resveratrol supplementation in the F0, F1 and F2 generations supporting a multigenerational effect. In addition, the level of DNA and RNA methylation (on 5mC and m6A, respectively) is increased in F1 offspring and associated with a change in the expression of enzymes involved in these epigenetic mechanisms, strongly suggesting epigenetic inheritance (Izquierdo et al., 2021). Future studies will be clearly required to understand if and how early nutritional interventions in mothers may exert beneficial effects in offspring and mitigate the long-term deleterious effects of adverse environmental exposure on late-onset AD.

Although most of the studies focused on the consequences of maternal environmental stress in the offspring, a growing body of evidence indicates that paternal preconception stress may also have long-term effects on the offspring. Studies in rodents indicate that paternal obesity induced by HFD alters the development of the 2-cell embryo. These early events have long-lasting consequences and decrease neurogenesis and memory, and are associated with epigenetic marks changes in adulthood, including hypermethylation of BDNF promoter, a neurotrophic factor that plays a key role in brain development and function (McPherson et al., 2015; Zhou et al., 2018). It has also been reported that repeated paternal stress (Harker et al., 2015, 2018), as well as paternal BPA exposure (Luo et al., 2017), lead to behavioural alterations, associated with changes in dendritic spines and brain organisation in the offspring. In addition, paternal stress during spermatogenesis leads to altered behaviour and DNA methylation patterns with increased level in the hippocampus of both male and female PND21 offspring (Mychasiuk et al., 2013). In mice, paternal cognitive spatial training improves cognitive performance in adult offspring and is associated with an increase in the hippocampal expression of synaptotagmin, a pre-synaptic protein. This was associated with elevation of histone acetylation at the promoter of synaptotagmin in the hippocampi of both fathers and offspring as well as in fathers sperm (Zhang et al., 2017). Thus, it would be interesting to delineate the role of paternal stress in animal models of AD and to determine whether both maternal and paternal exposure to stress would have a cumulative effect. Although it would more closely mimic what happens in humans, to our knowledge, this has never been reported in the context of AD.

As previously mentioned, a large majority of works have examined the consequences of maternal stress on male offspring. To date and to the best of our knowledge, only three studies look at the consequences of early-life environment on females, even though Alzheimer’s disease affects women preferentially. In the US, nearly 70% of AD patients are women, which can be explained by a longer life expectancy, but also by intrinsic biological differences, including brain organisation and function, age-associated loss of protection exerted by steroid hormones, higher inflammatory response, but also different susceptibility to genetic variations (for review Fisher et al., 2018). In addition, during early-development brain volume is higher in males, while the hippocampus is larger in females, pointing out to sexual dimorphism during a critical time-window (Kaczkurkin et al., 2019). Finally, although few studies have been reported in the context of AD, the use of mouse models led to discordant results in males and females showing the importance of studying the effects in the two sexes independently.

In conclusion, although the way by which perinatal epigenetic mechanisms act to program long-term memory and cognition remains to be clarified, it seems clear that the development of multi-omics strategies will allow molecular dissection of this fascinating new field of research. In particular, it will offer novel opportunities to identify early markers of AD and to develop novel preventive strategies to attenuate age-associated epigenetic alterations.
